# A Multi-Analysis of Children and Adolescents’ Video Gaming Addiction with the AHP and TOPSIS Methods

**DOI:** 10.3390/ijerph19159680

**Published:** 2022-08-05

**Authors:** Armita Khorsandi, Liping Li

**Affiliations:** 1 Injury Prevention Research Centre, Shantou University Medical College, Shantou 515041, China; 2 School of Public Health, Shantou University, Shantou 515041, China

**Keywords:** Video Game Addiction Scale, children and adolescents, the AHP method, the TOPSIS method

## Abstract

The video game market has become increasingly popular among children and adolescents in recent decades. In this research, we investigated the Video Game Addiction Scale (VGAS) for Chinese children and adolescents. We aimed to examine children and adolescents’ prioritization on the VGAS criteria and comparative analysis of the trend of video game addiction among them. A cross-sectional paper questionnaire study was conducted on 1400 Chinese students from grade 3 (9 years old) to grade 12 (18 years old). The respondents had to complete the socio-demographic information and the VGAS test. The VGAS characteristic was prepared in 18 criteria, which was the combination of the Video Game Addiction Test (VAT), Gaming Addiction Scale (GAS), and Revised Chinese Internet Addiction (CIAS-R). Eventually, the VGAS criteria prioritization was ranked methodologically through the Technique for Order Preference by Similarity to Ideal Solution (TOPSIS) method for each grade separately. Additionally, the Analytic Hierarchy Process (AHP) weighting technique was utilized to analyze the video game addiction of each grade under the four alternatives, individually. The results indicate that 3rd-grade students with some levels of addiction were the youngest who felt their life would not be fun without video games. Students in 5th grade with some levels of addiction were the youngest students who disclosed that their willingness to play video games is for forgetting their problems or feeling down. Moreover, they played video games more than before, thus, they did not sleep enough. Pupils of grade 6 reported that they played video games more than last semester. In their opinion, it is fair to play video games this much and does not need to reduce playing hours. Not getting enough sleep because of playing video games was seen in 7th graders as their first preference. 10th-grade students were the first to neglect to do their important responsibilities for playing video games. None of the 7th and 12th graders were somehow safe from video game addiction. In conclusion, playing video games can negatively affect studying, sleeplessness, getting far from society, and skipping important responsibilities for school students. Furthermore, the symptoms of video game addiction had seen at younger ages. These data provided insights for decision-makers to target effective measures to prevent children and adolescents’ video game addiction.

## 1. Introduction

With the advent of computers, tablets, cell phones, and progressing video games in the market, the willingness of children and adolescents to use those devices and play augmented reality video games have increased quickly. As such, in the last decades, the propensity to watch television has decreased [1]. Nowadays, computer games are available everywhere on any device, and due to the variety of computer games, everyone can find a favorite game for themself; this is a way to get addicted to playing video games. Video games are now an inseparable portion of children, adolescents, and adults. Recently, some games are connected to the internet, which allows gamers to play online video games with unknown adults or peers. Therefore, video game addiction includes internet gaming disorder. The over-usage of video games has been reviewed as a formal psychological disorder by the American Psychiatric Association (APA). Hence, the APA considered Internet Gaming Disorder (IGD) in the fifth revision of the Diagnostic and Statistical Manual of Mental Disorders (DSM-5) [2]. Overall, playing video games can be addictive.

In 2016, research for six European Union countries, from the School Children Mental Health Europe project, implied that 20% of the children play video games for more than 5 h a week [3]. In 2004, in Norway, a survey of the youth population revealed that they used the Internet almost 4.3 h per week, 35.8% were non-frequently and 49.6% were frequently using [4]. The first longitudinal study on violent video games was conducted with Chinese adolescents in 2019 [5]. From [6], we can get that playing some types of video games can enable the children to get far from the team working; they want to make their own things. They increase the violence against others by beating or killing them in the game to make things of their own. Children are increasingly getting addicted to video games. A group of children with the risk of addictive video games showed a shorter sleep duration, and most of the children played alone [7].

More than 85% of video games in the market contain some form of violence [8]. They promote the killing of people or animals, the use and abuse of drugs and alcohol, criminal behavior, disrespect for the law and other authority figures, sexual exploitation and violence towards women, racial slurs, foul language obscenities, and obscene gestures. These types of games reward immoral behavior that might lead gamers to suppose that an immoral attitude does not matter. These moral disengagement beliefs may be shifted from the virtual world to the real world after post-game. A study indicated that violent media, whatever it is, make people numb and indifferent to others’ pain and suffering [9]. In ref. [10], after comparing the levels of violence in video games, it concluded that more competitive games lead to more short-term aggressive behavior. For those in the maximum and medium blood conditions, the desire of using the character’s weapon is more often than in others [11]. A study in Taiwan showed that those groups of children and teenagers with high video game addiction had a higher level of hostility than others, as well as a lower level of academic achievements [12]. In addition, heart rate and blood pressure rise in children after playing video games [13].

Not all video games have a negative effect on children and adolescents. Mainly as a learning tool, educational games are used to teach subjects such as math or typing using basic game mechanics and thus stand out on most lists of best video games. Research has shown that the effectiveness of mobile video games in learning English language vocabulary can improve the student learning experience [14]. A study on patients with chronic stroke in Spain demonstrated the positive effect of virtual reality games on upper extremity functionality rehabilitation [15]. Moreover, some physical and movement games can reduce children’s obesity [16]. Spending extra time playing video games, even educational games, can get children and adolescents addicted to them. One of the evident negative consequences of extra and unscheduled playing of video games for children is the formation of mental patterns and identifying the game’s characters that are influenced by these computer games. Imitation on children’s appearance and behaviors is another harmful consequence of playing video games. As we see in society, many children and adolescents try to imitate and identify with these characters by buying clothes that are similar to the characters of the games and performing their behaviors, which also causes the children’s personalities to waver. Hence, we aim to analyze the issues that draw children and adolescents into playing video games.

**Figure 1:**
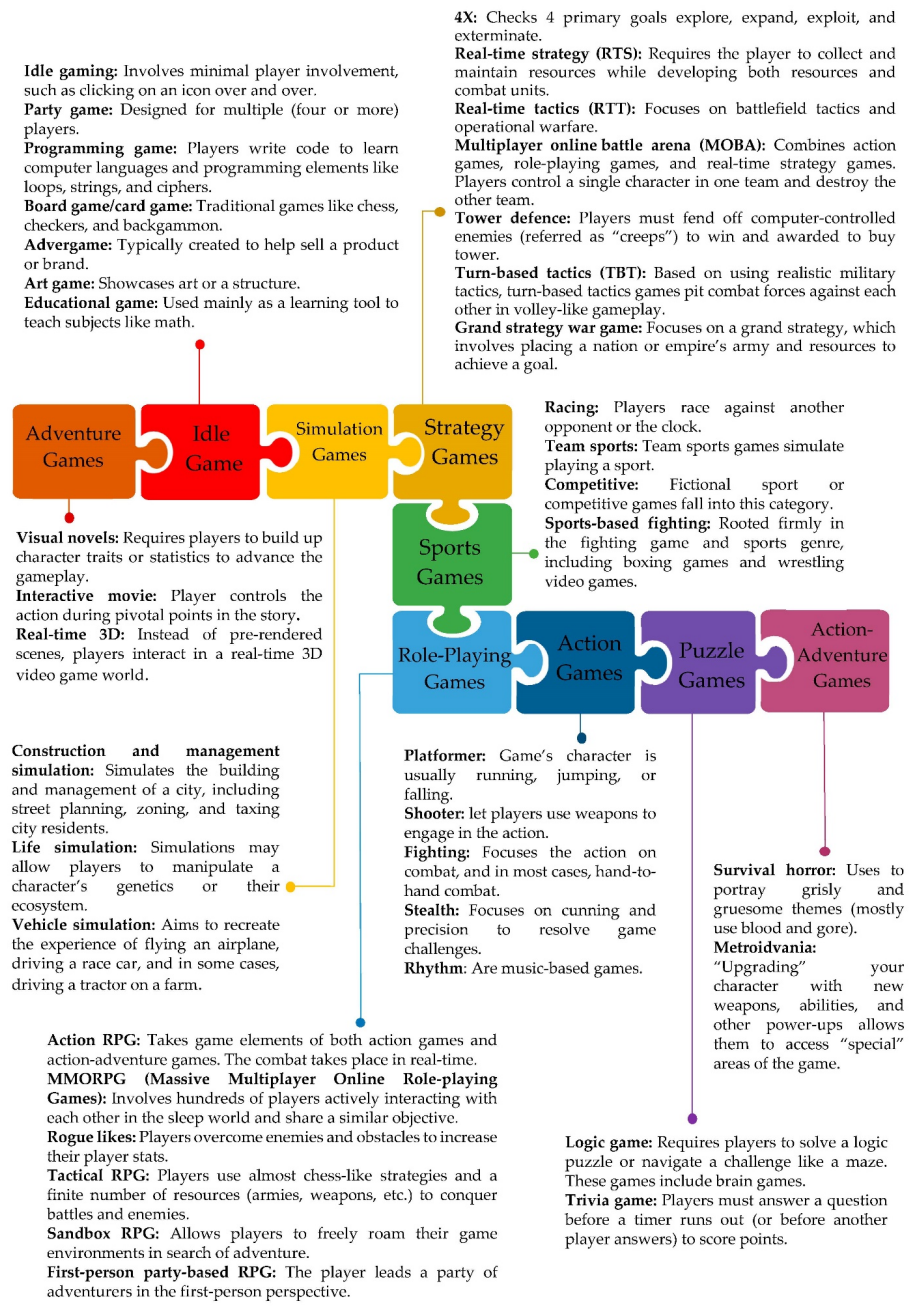
Different types of video games and some of their subgenres.

Different types of video games and some of their subgenres are briefed in Figure 1 [17].

Research on adolescents in Gorakhpur city revealed that 24.50% of adolescents were already addicted to playing video games [18]. A survey in Italy showed that during COVID-19, children and adolescents, mostly boys, got more involved in video games, with high rates of gaming disorder symptoms [19]. Adolescents grow up through sustained interactions within the family and then society. Some notable effects on reducing prosocial and increasing aggressive behavior were seen in children with exposure to video games [20]. Parents’ strategies to be friends with their children and adolescents and be beside them are the most impact factors in controlling them. Indeed, if their willingness to play video games is for a recreational motive or curiosity about something new, which is owing to their age, is not concerning in the first step, although after a while, playing video games will become a necessary part of their life [21]. The worries start when they replace playing video games with other entertainment, i.e., they replace their study time, family time, working-out time, and face-to-face conversation time to play the video game.

Adolescents at their puberty age are very fragile. A punishment or inappropriate behavior from others, violent behavior from a friend even by words, or being cut off from association due to an inability to do something, all can affect the mood of the children. This can persuade them to prefer socializing with people who do not know them and do not judge or condemn them, in order to find peace in their privacy. This isolation can be a flip to turn to computer games. One of the dangers of playing video games for children and adolescents is getting addicted to them. Mainly, parents cannot prevent their kids from playing such games. Therefore, the family will not be informed about their child’s work and their inexperienced child will be harmed in the virtual society. 

In this research, we investigated the video game addiction criteria in children and adolescents. “Video game addiction” is referred to as “gaming” in the psychopathology and psychiatry areas for mental taxonomies such as the ICD-11 [22], so in this research, we utilize the “gaming” label for “video game addiction”. An improved video game addiction test is adjusted to support our aim. A big range of school students were asked to participate in our survey and fill in the questionnaire. We used the Technique for Order Preference by Similarity to Ideal Solution (TOPSIS) method, as a technical method for the multi-criteria decision-making (MCDM) problem. The data were analyzed with the TOPSIS method to rank the criteria from the point of view of the video game players. In this method, it needs to give weights to the criteria. Although it can weight simply from the data, we use the analytic hierarchy process (AHP) method for calculating the weights for criteria to get an accurate result using TOPSIS. In our method, each question roles as a criterion. In the presented research, first, we analyzed the significant criteria from the point of view of the student from grade 3 (9 years old) to grade 12 (18 years old) separately, and then, we analyzed the criteria based on the alternatives.

Multi-decision analysis methods help decision-makers to identify criteria and factors that are related to the subject and weight them based on their significance [23]. One of the well-known tools for weighting the MCDM problems is the strong analytic hierarchy process (AHP) method, which was introduced by Saaty [24] for the first time. This method is accurate due to checking the consistency of the variables [25]. This method performs on paired comparison matrix between each pair of criteria. Once the pair-wise comparison matrix is implemented in the AHP method, the weighting process is executed [26]. In ref. [27], the AHP method was utilized to calculate the priority weight and rank the barriers to the development of renewable energy technologies in India.

One of the proven techniques to solve MCDM problems is the Technique for Order Preference by Similarity to Ideal Solution (TOPSIS) method, which was introduced by Hwang and Yoon [28]. In the process of the TOPSIS method, the criteria are ranked based on the similarity to the ideal solution. The strategic technique TOPSIS is distance-based, i.e., the alternative with a higher closeness coefficient to the best (positive) ideal solution is known as the preferred alternative [29]. Accordingly, The TOPSIS method sorts the criteria according to the closeness to the ideal solution by calculating the Euclidean distance from the ideal best. The TOPSIS method was utilized to rank different oilseeds to find the best suitable energy crop in Turkey [30]. In ref. [31], the TOPSIS method was used to rank the gasification type in thermochemical processes for different scenarios and find the top type.

However, many studies have worked on the negative effects of overusing computer games and most of the subjects were relevant to a small range of age. Besides, the detail of gaming tests was not analyzed and was just used to measure the level of the children’s gaming [4,32]. The main objectives of this research are listed as follows.

1. To study the extent of video game addiction among Chinese children and adolescents from grade 3 to grade 12.
2. To compare the trend of children and adolescents’ video gaming addiction and understand what reasons lead them to play video games and subsequently get addicted to video games, to take prevention measures.
3. To describe what factors of the VGAS were prioritized by children and adolescents, based on the alternatives they belong to, and conduct a comparative analysis on the trend of their criteria prioritization.
4. To identify the key priorities of VGAS criteria from the point of view of children and adolescents in grades 3–12 to avoid them getting addicted to playing video games.

The rest of this study is organized as follows. In Section 2, we have a discussion on participants and the procedure of our case study data that were used in this research. In addition, the AHP and TOPSIS methods are explained in detail. Section 3 is assigned to the different analyses of the VGAS test that were gained through the AHP and TOPSIS methods. In Section 4, we have further discussions. Eventually, Section 5 discusses the main findings.

**Hypothesis** **1** **(H1).** *Among the primary school pupils (grades 3, 4, 5, and 6) no cases are already addicted to playing video games.*

**Hypothesis** **2** **(H2).** *In Chinese school students, the incidence of getting addicted to playing video games begins from grade 7.*

**Hypothesis** **3** **(H3).** *Every 12th grader had some symptoms of addiction or is already addicted to playing video games.*

**Hypothesis** **4** **(H4).** *Identifying the level of addiction to playing video games among Chinese students from grade 3 (9 years old) to grade 12 (18 years old).*

## 2. Materials and Methods

### 2.1. Participants and Procedure

This study addressed the school students from grade 3 in primary school to grade 12 in high school. We have visited different schools in Shantou city of China in downtown and suburbs. Paper questionnaires were given to the students to fill in. The students had been selected randomly from various classes. The questionnaire took almost 20 min to complete. The purpose of the study was explained to the participants, and they had to provide consent to fill in the questionnaires. The poll was anonymous, and the information was confidential. The participants were asked not to consult with each other while they were answering the questionnaire. We conducted the survey in November 2021, and the data were collected for a period of four months. The questionnaire is listed in Appendix A.

Students consisting of 1400 cases who had participated in the survey, role as the decision-makers to evaluate the criteria. In this analysis, the VGAS questions are the set of the criteria, $Q_{i},\;i=1,\ldots ,18$, that anatomize for the alternatives, $DM_{j},\;j=1,\ldots ,4$, for four levels of gaming, including no symptom, at risk of addiction, have some levels of addiction, and already addicted. 

### 2.2. Ethics

This study was approved by the Ethics Committee of Shantou University Medical School (No. SUMC-2022-046). Additionally, the consent of the school’s student affairs office and counsellors were obtained. 

### 2.3. Methodology

**Figure 2:**
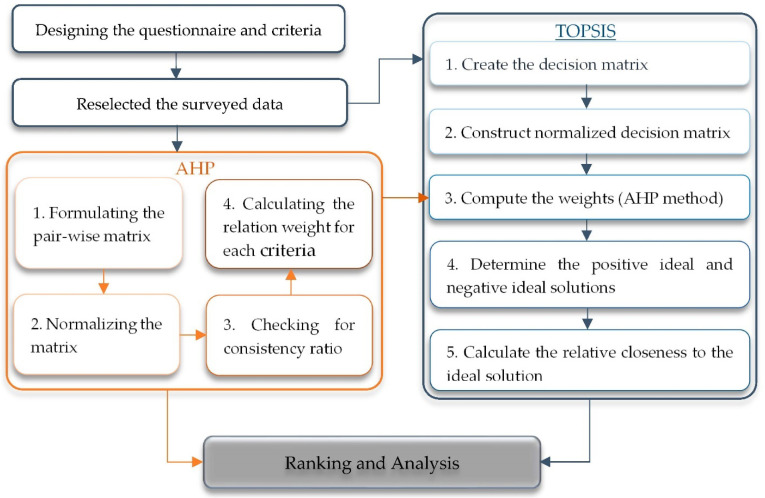
The methodology flowchart.

This study proposed a method to analyze the gaming situation of Chinese children and adolescents. To deal with this, the presented method is classified into four steps. The first step is dedicated to designing a proper survey and identifying the criteria. In the second step, the insufficient and incomplete data were removed. The third step is assigned to sort the criteria according to the decision-makers’ scoring, using the precise TOPSIS method. For getting better results, the criteria weights in the TOPSIS method are calculated by the AHP method. In the fourth step, the criteria are ranked and the results are compared. The proposed method is summarized in Figure 2, and the detail is discussed.

**Step 1. Designing the questionnaire and criteria.** In the present study, participants have been challenged on different scales. We assessed the gaming test for children and adolescents with socio-demographic information and the Video Game Addiction Scale (VGAS). Initially, the socio-demographic information about students such as age, gender, smoking and drinking status, number of friends, and the level of outstanding in class were collected. Subsequently, the students answered the VGAS questions. First, the level of children’s and adolescents’ gaming was measured. As 14-item Video Game Addiction Test (VAT) [33] did not adequately support all our purpose criteria, to evaluate the gaming status of children and adolescents, we designed the 18-item Video Gaming Addiction Scale (VGAS). The combination of the subscription of VAT, 7-item Gaming Addiction Scale (GAS) [34], and 19-item Revised Chinese Internet Addiction Scale (CIAS-R) [35] with DSM-5 Likert scales [36], “never”, “rarely”, “sometimes”, “often”, and “always” created our 18-item VGAS test. The VGAS questions create the criteria of our method. By following the previous test scoring, the addiction level is divided into 4 levels [33]. A score of 0–22 implies that there is no symptom of addiction in their video gaming behavior, 23–45 shows they are at risk of getting addicted to video games, 46–67 is an alarm that the child have some levels of addiction, and 68–90 represents gamers who are already addicted to video games. Here, we define online and offline video games as computer games and hand-held games such as mobile, computer, and tablet devices.

**Figure 3:**
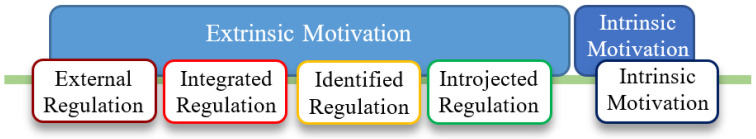
Extrinsic and intrinsic motivations.

Video gaming motivation is a complex matter that includes intrinsic and extrinsic motivation components. The extrinsic motivation components are included integrated regulation, identification regulation, introjected regulation, and external regulation. Internal, personal feelings, and environmental forces influence intrinsic motivation. In addition, friends and family can belong to the extrinsic motivation category (Figure 3).

**Step 2. Reselected the surveyed data.** To avoid adding incomplete and insufficient answers to our method, the questionnaire were reviewed. The criteria for removing the unsuitable questionnaire are as follows. Choosing repeated options to all multiple-choice questions, filling the questionnaires very quickly, leaving a question blank in the scoring test, and choosing two answers for single-answer questions.

**Step 3. The TOPSIS method.** The compromise ranking TOPSIS method is adopted as an appropriate method to solve the MCDM problem that is non-commensurable and contradictory criteria. The procedure of the TOPSIS method is presented as follows. In this comparison benchmarking analsis, the set of criteria $Q_{i},\;i=1,\ldots ,n$, is compared with respect to a set of alternatives $DM_{j},\;j=1,\ldots ,k$, under a group of decision-makers. The TOPSIS method calculates the shortest distance from the ideal best solution and the farther distance from the ideal worst solution. Supposed that T is an MCDM matrix with k alternatives on rows and n criteria on columns (Equation (1)). The values of the rows are the summation of the normalized decision-maker’s performance rating to the criteria.

(1) $$T={\left\{t_{ji}\right\}}_{k\times n}=\left[\begin{array}{ccc} \begin{array}{cc} t_{11} & t_{12}\\ t_{21} & . \end{array} & \begin{array}{c} \cdots \\ \ldots \end{array} & \begin{array}{c} t_{1n}\\ . \end{array}\\ \begin{array}{cc} \vdots & \;\vdots \end{array} & \ddots & \vdots \\ \begin{array}{cc} t_{k1} & . \end{array} & \cdots & t_{kn} \end{array}\right]$$

The TOPSIS method is based on a normalized decision matrix. For normalizing the decision matrix, each typical element of the T matrix is divided by the square summation of the elements in the related column with Formula (2):

(2) $$NT_{ji}=\frac{t_{ji}}{\sqrt{\sum_{j=1}^{k}t_{ji}{}^{2}}},$$

After calculating the normalizing decision matrix, the weighted normalized decision matrix should compute with Equation (3) as follows:

(3) $$NW_{ji}=w_{i}\frac{t_{ji}}{\sqrt{\sum_{j=1}^{k}t_{ji}{}^{2}}},$$

where $w_{i}$ is the importance degree (weight) for the i-th criteria. To get more accurate results in this research, we used the precise AHP method to compute the weights (that will be explained later). Then, it needs to determine the positive (best) ideal solution (PIS) and the negative (worst) ideal solution (NIS). The ideal best and ideal worst solution formulas are defined as follows [37]:

(4) $$\begin{array}{c} PIS=\underset{j}{\max}\{NW_{ji},\;i=1,\ldots ,n\}=I_{i}^{+}\\ NIS=\underset{j}{\min}\{NW_{ji},\;i=1,\ldots ,n\}=I_{i}^{-} \end{array}$$

Next is to compute the separation distances $db_{i}$ and $dw_{i}$ for i-th criteria from the ideal best and ideal worst values through Equation (5), respectively. For this issue, the Euclidean distance method is employed.

(5) $$\begin{array}{c} db_{i}=\sqrt{\sum_{j=1}^{k}\left(NW_{ji}-I_{i}^{+}\right)},\;\;i=1,\ldots ,n,\\ dw_{i}=\sqrt{\sum_{j=1}^{k}\left(NW_{ji}-I_{i}^{-}\right)},\;\;i=1,\ldots ,n, \end{array}$$

Eventually, the relative closeness to the ideal solution for each criterion is calculated by Formula (6):

(6) $$R_{i}=\frac{dw_{i}}{db_{i}+dw_{i}},\;\;i=1,\ldots ,n.$$

Indeed, a higher value of $R_{i}$ indicates the lower ranking order for criteria. A lower $R_{i}$ means the criterion has less distance to the ideal solution.

**The AHP method.** After collecting the data and scaling through the improved questionnaire, the output was inserted into the AHP method with respect to the proper weighting of the TOPSIS model. The first step to weight the criteria through the AHP method is to build the pair-wise comparison matrix. According to the hierarchical structures, the decision-maker can scale the system by scoring the criteria. The positive pair-wise comparison matrix [X] is formulated based on the decision-makers rating for n criteria in Equation (7):

(7) $$X={\left\{x_{ij}\right\}}_{n\times n}=\left[\begin{array}{ccc} \begin{array}{cc} 1 & x_{12}\\ x_{21} & 1 \end{array} & \begin{array}{c} \cdots \\ \ldots \end{array} & \begin{array}{c} x_{1n}\\ . \end{array}\\ \begin{array}{cc} \vdots & \;\vdots \end{array} & \ddots & \vdots \\ \begin{array}{cc} x_{n1} & . \end{array} & \cdots & 1 \end{array}\right]=\left[\begin{array}{ccc} \begin{array}{cc} 1 & \frac{s_{1}}{s_{2}}\\ \frac{s_{2}}{s_{1}} & 1 \end{array} & \begin{array}{c} \cdots \\ \ldots \end{array} & \begin{array}{c} \frac{s_{1}}{s_{n}}\\ \frac{s_{2}}{s_{n}} \end{array}\\ \begin{array}{cc} \vdots & \;\vdots \end{array} & \ddots & \vdots \\ \begin{array}{cc} \frac{s_{n}}{s_{1}} & \frac{s_{n}}{s_{2}} \end{array} & \cdots & 1 \end{array}\right],$$

where $x_{ji}=\frac{1}{x_{ij}}$, for i≠j and $x_{ij}=1,$ for i=j and $s_{n}$ is the total score that is attained for each criteria. The matrix’s number of criteria is n and $x_{ij}$ is equal to the division of the score given by the decision-maker to i-th criteria by j-th criteria, which is called the comparison value. The judgment scale that was used in this research is the original Saaty scale [24]. The scores are turned on a scale of 9 according to the Saaty scores, to formulate a pair-wise comparison.

After that, the summation of the scores is calculated for each criterion. For normalizing the pair-wise comparison matrix, each element of the matrix, $x_{ij}$, is divided by the related summation score of j-th column value with Formula (8):

(8) $$N_{i}=\frac{x_{ij}}{\sum_{j=1}^{n}x_{ij}},\;\forall i,j.$$

The next step is to compute the average for the individual variants. To determine the respective weight values for each criterion, $w_{i}$, the average of the normalized matrix, $N_{i}$, is calculated for the row with Formula (9). The summation of the weight must be equal to 1.

(9) $$w_{i}=\frac{\sum_{i=1}^{n}N_{i}}{n},\;\overset{N}{\underset{i=1}{\sum }}w_{i}=1.$$

**Check for consistency ratio (CR)**: To make sure that our weighing values are correct, the level of the consistency of the pair-wise comparison matrix must be checked. The consistency index (*CI*) can be computed as follows:

$$CI=\frac{L_{\max}-n}{n-1},$$

$$L_{\max}=\overset{n}{\underset{i=1}{\sum }}(w_{i}\times \overset{n}{\underset{j=1}{\sum }}x_{ij}).$$

where $L_{\max}$ represents the highest eigenvalue of the matrix. *CI* = 0 implies that the pair-wise comparison matrix is perfectly consistent. If *CI* > 0, then the level of inconsistency should be examined by the Saaty scale [24] with the following formula:

$$CR=\frac{CI}{RI}$$

where *CI* is the consistency index, and *RI* is the random consistency index which is proposed by Saaty [38] in Table 1. 

In the AHP, if the CR is less than 0.1 then the consistency is accepted, otherwise, it will be rejected. The weights that are gained by the AHP method will be used as the weight score that is required for the TOPSIS method.

**Step 4. Ranking and Analysis.** As we have three groups of analysis, this step is discussed in Results and Discussion sections. 

## 3. Results

### 3.1. Case Study

As mentioned in Section 2.1., a total of 1400 students took part in our survey, consisting of 120–150 participants for each grade. The data was reselected based on the Step 2 description, and we kept almost 100 cases per grade to make a balanced result. According to the COVID-19 situation, accessing more students was impossible.

In this research, we aimed to rank the criteria from the students’ point of view for each grade. In addition, we investigated the top criteria from the point of view of the alternatives per grade. For this purpose, the TOPSIS and AHP methods were utilized. We used the AHP method for weighting criteria because of its accuracy, due to checking the consistency of the variables, to make sure the weights are correct. The TOPSIS method is one of the most well-known and common techniques for solving MCDM problems to rank the alternatives. Therefore, it was reliable for this research and could cover the results adequately for the alternatives.

In this study, to get the real answer from the point of view of the children and adolescents, the student’s scores were made into the pair-wise comparison matrix in the AHP method and the decision matrix in the TOPSIS method. The questionnaires were given to the students to score all the questions from 1 to 5, using a 5-point Likert scale. The scores were summed together, and based on that, students were placed in one of the four alternative groups DM1–DM4.

**AHP method.** To make the pair-wise comparison matrix, the total score for each question was calculated and rescaled from 1 to 9 for the Saaty scale. Since the values of the pair-wise comparison matrix are the ratio of each criterion over the other criterion, we obtained these values by dividing them by each other and gained the 18 × 18 pair-wise comparison matrix in Equation (7) with $x_{ji}=\frac{1}{x_{ij}}$, for i≠j and $x_{ij}=1,$ for i=j. The weights of the AHP were calculated by Equation (9). The results of the AHP method are displayed in Table 2, Table 3, Table 4 and Table 5.

**Figure 4:**
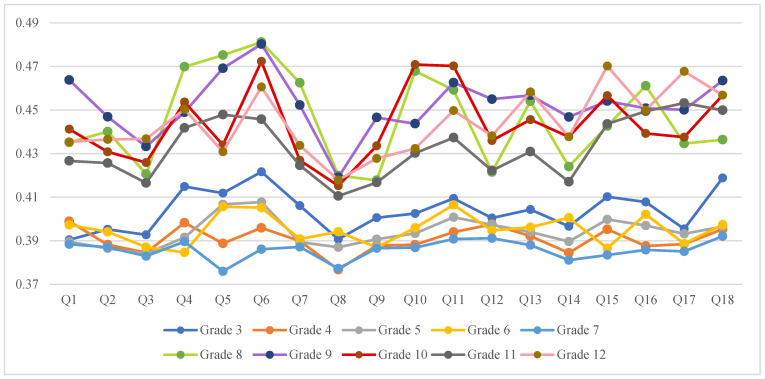
The VGAS criteria analysis for grades 3 to grade 12 with TOPSIS method.

**TOPSIS method**. We performed two different analyses by ranking alternatives and criteria, which are discussed in Section 3.3 and Section 3.4. According to the TOPSIS method, the decision matrix in our study is a 4 × 18 matrix in Equation (1) consisting of four alternatives DM1–DM4 on rows and 18 criteria on columns. Using Equations (2) and (3), with the weights that were achieved through the AHP method, we calculated the normalizing decision matrix. With the aid of Equation (4), the ideal best and ideal worst were computed. Here, to rank the alternatives (DM1–DM4), the separation distances and ratio should be calculated with Equations (5) and (6) on rows. The results of the ranking of the alternatives are displayed in Table 6. On the other side, to find the students’ point of view about prioritizing criteria, we ranked the criteria by calculating the separation distances and ratio on the columns with the concept of Equations (5) and (6). The results of the criteria ranking are displayed in Figure 4 and Table 7.

### 3.2. The VGAS Criteria Analysis with the AHP Method

In this section, we analyzed the VGAS criteria ranking with the weights of the AHP method based on four alternatives for each grade separately.

The AHP method is using for weighting the criteria, and finding the best and the worst values. We aimed to analyze the prioritized criteria that were chosen by children and adolescents from the point of view of the four alternatives, “no symptom of addiction” (DM1), “at risk of getting addicted to video games” (DM2), “have some levels of addiction” (DM3), and “already addicted to video games” (DM4) separately. Here, each alternative works as an independent group. The rows in Table 2, Table 3, Table 4 and Table 5 are the results of the AHP weights on independent alternatives. For instance, the values of row DM1 in Table 2, Table 3, Table 4 and Table 5 indicate the weights of the criteria with respect to the DM1 scores. These values were calculated individually for grades 3 to 12 and displayed in Table 2, Table 3, Table 4 and Table 5. The “weights” row implies the criteria weights that were gained by the AHP method from the point of view of the total pupils in each grade. 

**Grade 3.** As we can see in Table 2, in 3rd grade, for those who were the students without gaming addiction symptoms (DM1), Q7 and Q10 were a priority; they looked forward to the next time they could play video games (Q7), and others unsuccessfully tried to reduce their video game playing hours (Q10). For at-risk pupils, Q8 and Q10 were a priority, i.e., they thought they should play less video games (Q8), but others could not stop them from playing the video game (Q10). Those who had some levels of addiction felt that their life would be no fun without playing video games (Q18), and others could not successfully prevent them from playing video games (Q10). Although, no cases of gaming addiction were found among 3rd-grade students, this could be a sign of their addiction to video games that may occur in the future. It needs expert decision-makers to consider deeply this problem.

**Grade 4.** The importance of the criteria from the point of view of 4th-grade students was different. Those who did not have any symptoms of addiction preferred to play video games instead of spending time with others (Q4). Those who were at risk of gaming argued with their family over their time-spending on video games (Q3), while they believed that they should play less video games (Q8). For students with some levels of addiction, others tried to reduce their playing time but failed (Q10).

**Grade 5.** Those 5th graders without symptoms of addiction suggested that they should reduce their video game playing hours (Q8), although they tried several times to reduce their playing time but failed (Q9). Also, at-risk pupils similarly believed in reducing their playing video game hours (Q8). Those students who had some symptoms of gaming addiction stated that they did not sleep enough because of playing video games (Q5), as well as they thought about playing video games all day long (Q6). Additionally, they reported that they were spending more time playing video games than last semester (Q15). They disclosed that their willingness to play video games is due to forgetting their problems or feeling down (Q14).

**Grade 6.** Students with no symptoms of addiction in grade 6 thought that they should play less video games (Q8). From the point of view of at-risk 6th graders, although they knew they should play less (Q8), they argued with others to spend more time playing video games (Q3). For students with some levels of addiction, Q14 preceded other criteria. They disclosed that they play video games when they feel upset or have some problems.

**Grade 7.**Table 4 shows that, unfortunately, all the 7th graders were somehow faced some symptoms of addiction to video games. The at-risk student group understood that they should play less video games (Q8). Pupils with some levels of addiction affirmed they play games more than they intended (Q2). Those who had been already addicted behaved relatively similarly to DM3. In addition, they reported some arguments with their families to spend more time playing video games (Q3). They always look forward to the next time playing video games (Q7). They felt that playing extra video games affected their studies negatively (Q17).

**Grade 8.** As shown in Table 4, the prioritization of criteria for those students of grade 8 with no symptoms of addiction was Q8 and Q14. They believed that they should play less video games (Q8), but they expressed the reason for playing video games is to forget problems or feeling down (Q14). However, students that were at risk of addiction suggested that they had to reduce their playing hours (Q8), they had a controversy with others to play more video games (Q3). Graders with some levels of gaming in grade 8 showed different behavior. They admitted that stopping video gaming is difficult for them (Q1), and for having extra video game playing hours (Q2) they had to argue with their family (Q3). The addicted gamers selected Q2, Q3, Q8, and Q14 criteria. The positive point is that they believed they should play less games (Q8).

**Grade 9.** 9th-grade students who did not have any symptoms of addiction agreed that they play more video games than they scheduled (Q2), likewise, they usually play video games because they feel down or have some problems (Q14). For at-risk students to gaming, in addition to Q2 and Q14, they reported some contention with others to play more video games (Q3), although they believed that they should play less (Q8). Those who had some levels of addiction behaved similarly to DM1. For those who were addicted to playing video games in grade 9, we saw some differences in the ranking criteria. They finished their daily responsibilities in a rush to play video games (Q12). However, they thought they played extra video games (Q2), and their attempt to play less video games was unsuccessful (Q9).

**Grade 10.** In grade 10, the gaming criteria trend for those who did not show any symptoms of addiction was new. They reported that they neglected their important activities such as school assignments to play video games (Q13). Moreover, they expressed some arguments with their family about playing more video games (Q3). Students that were at risk of getting addicted prioritized Q2, Q3, Q8, and Q14. They tended to play more video games than their intention (Q2), as well as having discussions with their family about more video gaming hours (Q3). It was a surprise seeing that they believed they should play less video games (Q8). However, they have stated they play video games because of some problems or feeling down (Q14). Students with some levels of addiction in the 10th grade had the same opinion as DM2 in this grade, except that they did not believe they need to cut back on playing video games. Unfortunately, addicts to video games stated that they spend lots of time playing video games (Q2) because it is difficult to stop playing them (Q1). They even preferred playing video games instead of spending time with friends (Q4). Moreover, they reported a lack of sleep due to playing video games (Q5). Furthermore, they did their work in a rush to have more time to play video games (Q12), as well as neglecting their important activities such as homework (Q13). In one word, playing video games is a priority over everything for them.

**Grade 11.** According to Table 5, students who did not show any symptoms of gaming selected Q13 as the most paramount criteria. They neglected their important activities such as school homework to play video games. However, students that were at risk of addiction believed they should play less video games (Q8). They liked to play video games when they feel down or have some problems (Q14). They said they played extra video games (Q2) and disputed with others to spend more time playing (Q3). Similarly, for students with some levels of addiction Q2, Q8, and Q14 took precedence over other criteria. Criteria Q2, Q4, Q14, and Q15, were preferred for gamers. They reported that they preferred to play video games instead of spending time with family or friends (Q4). Further, they admitted to spending more time playing video games (Q2). They accounted that they spent more time playing video games than before (Q15), mostly when they feel down or had some problems (Q14).

**Grade 12.** Unfortunately, in the 12th grade, all the students had some symptoms of addiction to video games or were at risk of it. For the students at risk of gaming, although they have extra time playing video games (Q2) and argued with others over playing time (Q3), they believed that they should reduce their video game playing hours (Q8). For those who had some levels of addiction, they confessed to extra playing (Q2). Additionally, they discussed with their family to spend more time playing video games (Q3). They even neglected important activities to play video games (Q13). The addicted students reported that they altercated with others for having more time playing video games (Q3). However, they believed that playing video games caused negative effects on their study (Q17), they usually played video games when they feel down or have a problem (Q14). They faced some physical discomforts because of playing video games (Q16). Furthermore, they felt that their life is not happy without playing video games (Q18).

### 3.3. The VGAS Criteria Analysis on Alternatives Using the TOPSIS Method

In this section, we employed the TOPSIS method to compare the four alternatives (DM1–DM4) against the criteria for each grade separately. For getting a more accurate result, the weights of the TOPSIS method are calculated with the AHP method. In this analysis, the alternatives are ranked based on their proneness to video game addiction. The separation distances, ratio values, and the ranking of the alternatives are classified in Table 6 for each grade.

Table 6 implies that for grade 3, DM2 > DM3 > DM1, i.e., among the students of grade 3, those who were at risk of gaming were the most susceptible students to get addicted to gaming, and those who had some symptoms of gaming were ranked second. This order of ranking was the same for grades 4 and 6, but in grade 5, the ranking of the alternatives was DM2 > DM1 > DM3. 

For middle school pupils (grades 7, 8, and 9), those who were at risk of gaming (DM2) were the most susceptible pupils to become addicted to video games, and those who had some symptoms of gaming (DM3) stand in the second place, however, they had some differences on 3rd and 4th places.

The ranking of the alternatives for high school graders was the same in grades 10, 11, and 12 as DM2 > DM3 > DM4 > DM1. For them, those who were at risk of gaming were the most susceptible students to gaming, and those who had some symptoms of gaming stand in the second rank.

As we have seen in Table 6, the DM2 group is the most sensitive and susceptible group to gaming. It shows that contrary to popular beliefs that may think those who have some symptoms of video game addiction are more exposed to gaming, this research proved that those who do not have symptoms of gaming but are at risk of becoming addicted to video games are more susceptible to gaming than other groups.

### 3.4. The VGAS Criteria Analysis with the TOPSIS Method per Grade

We conducted an analysis of the criteria of the VGAS using the TOPSIS method on a total of 18 criteria that are associated with video game addiction. After calculating the weights of the criteria with Equation (9) with the AHP method, the relative closeness criteria to the best ideal solution was computed by Equation (6). As mentioned in Section 3.1, in this analysis, after calculating the ideal best and ideal worst with Equation (4), we ranked the criteria by calculating the separation distances and ratios with the concept of Equations (5) and (6). The aim is to check the children’s and adolescents’ preferences towards the criteria. The results are displayed in Figure 4 and Table 7.

Figure 4 indicates that criteria Q1, Q8, and Q3 are the top three criteria that were selected by grade 3, i.e., although they said that it is difficult to stop playing video games (Q1) and they argue with others to spend more time playing video games (Q3), they believed they should play less (Q8). Grade 4 selected Q8, Q14 (playing video games because of feeling down or forgetting the problems), and Q3 as the top three criteria. Q3, Q2, and Q8 are the three priority criteria that were chosen by grade 5. Although they argued with their family about having more video games (Q3) and spent extra video gaming (Q2), they believed that they should play less video games (Q8). Grade 6 made a big change in the scoring criteria; Q4, Q9, and Q15 were the top three criteria for them. Although, their top priority was to play video games instead of spending time with others (Q4), they attempted to spend less time playing video games but failed (Q9). They found that they play video games more than before (Q15).

Although getting not enough sleep because of playing video games (Q5) is the top factor that was opted by the 7th-grade students, lesser video gaming (Q8), and playing video games because of feeling down (Q14) stand in second and third place of priority, respectively. These values are changed for grade 8 by prioritizing Q9, Q8, and Q3. The 8th-grade students, as the first top criteria, struggled to spend less time playing video games although they failed (Q9). Playing less video games (Q8) was their second target, but they had disputed with family over having more playing hours (Q3). For 9th grade students, Q8, Q3, and Q10 are the top three criteria. Although they were aware that they should reduce their playing hours (Q8), on the other hand, they wanted to have more video gaming (Q3). In addition, others tried to reduce their video gaming hours, but they had not succeeded (Q10).

For high school students, the priorities were a bit different. The main goal of all of them was to reduce their video gaming hours. For 10th grade pupils, Q8, Q3 (altercating with others to spend more time playing video games), and Q7 (looking forward to the next video game playing turn) were before other criteria. For grade 11, the trend was similar. Q8, Q3, and Q9 were the 11th-grade students’ priorities. The 12th-grade pupils prioritized Q8 as the top choice. They unsuccessfully tried to spend lesser playing (Q9) which was in second place. Also, they reported less sleep because of playing video games (Q5) as the third choice.

The trend of ranking the criteria is almost the same for the 3rd and 4th graders. The sorting of the criteria trend for the students in grades 5 and 6 is not much similar. However, the ranking of the criteria trend for the 5th and 7th graders is nearly the same. The sixth graders, who were about to finish elementary school, behaved differently in the criteria ranking than students in their age group (grades 5 and 7). It implies that they may become too immersed in playing video games. As shown in Figure 4, the only grade that thought reducing the level of video game timing is not necessary was grade 6.

Although the 7th and 8th-grade pupils had few disagreements on a few criteria, they followed a somewhat similar trend in the ranking criteria. As 7th graders still followed primary students’ demeanor, perhaps it can be derived that the importance of criteria has been changed, as the junior middle school students, when they grow with senior middle school students. The pupils of grades 8 and 9 did not show the same behavior in the ranking criteria, while graders 10, 11, and 12 acted very similarly except in two criteria.

The criteria ranking was quite different among middle school students (grades 7, 8, and 9). They seemed to be in a particular conflict over playing video games. Since they are at a risky age, their video game conduct needs to be further explored.

All the criteria in the VGAS test with the AHP weighting were included in the TOPSIS method for ranking priorities analysis in Table 7.

Students in grade 3 who found it difficult to stop playing video games (Q1) had Q6, Q18, and Q4 as their last choices. The 4th graders who were given second place to Q2 did not tend to play video games instead of being with their friends (Q4). For them, it was easy to stop playing video games (Q18), as well as the fact that they spent enough time on their daily responsibilities (Q12). For pupils in grade 5, even though they reported playing the extra video games (Q2), they did not take their sleep time for playing video games (Q5). Moreover, they did not count the moments playing video games again (Q6). In addition, they have not reported getting upset or frustrated when they do not have access to video games (Q11). The most insignificant criteria for the 6th graders were quite similar to the 5th graders.

Pupils of grade 7 who often did not get enough sleep due to playing video games (Q5), had a higher risk of feeling alone or depression, as Q14 was their third choice. For them, the Q11, Q12, and Q18 criteria were ranked last. They did not get sad or irritated when they could not access video games (Q11), as well as they did not rush in their daily work due to having more time to play video games (Q12). Moreover, the fun of their life was not limited only in playing video games (Q18). Students of grade 8 did not prefer playing video games over being with friends (Q4). Q5 and Q6 were the criteria with the lowest importance for them. The 9th graders had the same choices as the 8th graders on ranking Q5 and Q6. Also, they claimed they can stop playing video games anytime that they like (Q1).

For 10th graders who looked forward to playing video games (Q7), no one had tried to help them reduce their playing time (Q10). Students in grades 11 and 12, who had tried to play less video games but did not succeed (Q9), claimed that playing video games did not have a negative effect on their studies (Q17). Students of grade 12 who slept less to have more time playing video games (Q5), reported they did not play more than last semester (Q15).

In general, Q8 ranked as the topmost criteria by children and adolescents except for grade 6. Q3 ranked as the second most important criteria and Q6 as the poorest criteria by children and adolescents. Students who thought they should play less video games (Q8) were more likely to argue with others over playing more video games (Q3). Thinking about playing video games all day long (Q6) was their last choice except for grade 7. Middle school students were the most challenging pupils, as they had no similarity in ranking the criteria.

## 4. Further Discussion

Based on the definition of VGAS, 5.63% of the students in grade 12 had suffered from gaming addiction, 22.53% had some levels of addiction, 71.83% were at risk of addiction, and no student had no symptoms of gaming addiction. This is somewhat worrying that almost all the grade 12th students showed a symptom of gaming addiction. For grade 11, these numbers were 2%, 24%, 72%, and 2% for already addicted, have symptoms of addiction, at risk of addiction, and free of gaming addiction, respectively. The gaming rate in grade 10th was 2%, 26%, and 71% for addicted students, have some levels of addiction, and at risk of addiction, respectively. Only 1% of the students did not have any symptoms of addiction.

The students of grade 9 had experienced another rate of gaming. Although, the rate of those who did not have any symptoms of gaming was 3%, almost 5% of the students were already addicted to video games. This is more than students in grades 10, 11, and 12. Of the rest of the students, 25% had some symptoms of addiction, and 67% were at risk of gaming. Students in grade 8 showed promising video gaming behavior. Among them, 7.93% did not have any symptoms of gaming addiction, and 79.36% were at risk of addiction. Only 11.11% of them had some symptoms of addiction, and 1.58% were already addicted to playing video games. The rate of gaming addiction results was concerning for grade 7. There was no student who did not have some symptoms of addiction, i.e., all of them were involved in gaming somehow. Almost 89% of them were at risk, 10% had some symptoms of addiction, and 1% of the total were already addicted to playing video games.

The highest grade that the students were not getting addicted to playing video games was grade 6. Almost 18% of 6th graders reported that they did not have any symptoms of gaming addiction. A total of 73% reported that they are at risk of addiction, and 9% had some levels of gaming addiction. In the 5th grade, only 6.1% of the students had some levels of addiction, 76.83% were at risk, and 17.07% of the total had no symptoms of gaming addiction. The addiction trend is lower among grade 4 students. A total of 11.32% of them did not have any symptoms of addiction, 67.92% were at risk, and 20.75% had some symptoms of addiction. The level of gaming in grade 3 is at its lowest value. A total of 12.12% of the students had no symptoms of addiction, 75.75% were at risk, and only 12.12% had some symptoms of addiction to video games (Table 8).

## 5. Conclusions

In this research, a novel VGAS, the combination of VAT, GAS, and CIAS tests, has been proposed based on four alternatives for different levels of addiction to video games. Firstly, the AHP method was used as the underlying model to weight the criteria and analyze the student’s criteria prioritization under each alternative individually. Then, the TOPSIS method was adopted to rank the criteria. The results implied that among the primary school students (grades 3, 4, 5, and 6) no case of gaming addiction was found. The 6th grade was the only grade that thought it was fair to play video games this much and did not need to reduce their playing hours; they were prone to get addicted to playing video games. The first addicted students were seen in grade 7. They confessed that they played video games longer than they intended. In contrast, no pupils in grade 7 and grade 12 were free of gaming addiction. Moreover, grade 7 as the first grade of middle school, and grade 12 as the last grade of high school, reported not having enough sleep because of playing video games. Furthermore, students with an addiction to video games in grades 7 and 12 were the only students who believed that playing video games negatively affected their studies. It seems that 7th and 12th graders have some similar attitudes towards playing video games.

Playing video games because of feeling down or forgetting problems, seriously started in grade 8 students; this needs psychologists to check whether this attitude occurs after students start getting addicted to playing video games in grade 7. Middle school pupils did not have any similarity in prioritizing the criteria. Neglecting important activities was seen poorly just among high school students. Reducing playing time was a top priority for high school graders. The number of students in grades 10 and 11 who did not show any symptoms of gaming was only 1% or 2% of the total. Instead, the number of those who were at risk of gaming was around 72%. Overall, students become more inclined to play video games and subsequently become more addicted to playing video games as they get older. As previous studies have not deeply concentrated on VGAS criteria, thus, these results can help decision-makers to have a better understanding of children and adolescents’ preferences criteria in playing video games and take measures to reduce their video game playing hours.

In our future work, we plan to find out the fundamental reasons that encourage children and adolescents to play video games. The causes can be their friends, society, their feeling, or even family.

## Figures and Tables

**Table 1:** Ratio index.

Number of Attributes	1	2	3	4	5	6	7	8	9	10
*RI*	0.00	0.00	0.52	0.89	1.11	1.25	1.35	1.40	1.45	1.49

**Table 2:** The weight and the ranking of VGAS criteria for grades 3 and 4.

	Q1	Q2	Q3	Q4	Q5	Q6	Q7	Q8	Q9	Q10	Q11	Q12	Q13	Q14	Q15	Q16	Q17	Q18	Sum	CI
Grade 3																				
DM1	0.048	0.054	0.060	0.066	0.054	0.048	**0.072**	0.054	0.066	**0.076**	0.048	0.048	0.054	0.060	0.048	0.048	0.048	0.048	1	0
DM2	0.049	0.057	0.068	0.049	0.043	0.041	0.057	**0.081**	0.061	**0.072**	0.046	0.052	0.055	0.059	0.049	0.045	0.045	0.051	1	0
DM3	0.030	0.045	0.050	0.061	0.050	0.061	0.058	0.061	0.055	**0.072**	0.055	0.050	0.058	0.048	0.061	0.050	0.058	**0.076**	1	0
DM4	0.000	0.000	0.000	0.000	0.000	0.000	0.000	0.000	0.000	0.000	0.000	0.000	0.000	0.000	0.000	0.000	0.000	0.000	--	--
Weight	0.046	0.055	0.065	0.053	0.045	0.045	0.059	0.075	0.060	0.070	0.048	0.052	0.055	0.057	0.051	0.046	0.063	0.055	1	0
Grade 4																				
DM1	0.048	0.064	0.064	**0.089**	0.056	0.048	0.048	0.064	0.056	0.050	0.056	0.048	0.048	0.048	0.064	0.048	0.048	0.048	1	0
DM2	0.044	0.055	**0.072**	0.050	0.038	0.042	0.050	**0.084**	0.067	0.054	0.050	0.060	0.047	0.067	0.055	0.042	0.064	0.059	1	0
DM3	0.040	0.052	0.054	0.061	0.050	0.061	0.062	0.064	0.048	**0.072**	0.059	0.047	0.054	0.055	0.054	0.050	0.061	0.057	1	0
DM4	0.000	0.000	0.000	0.000	0.000	0.000	0.000	0.000	0.000	0.000	0.000	0.000	0.000	0.000	0.000	0.000	0.000	0.000	--	--
Weight	0.043	0.055	0.066	0.056	0.043	0.048	0.054	0.076	0.061	0.059	0.053	0.055	0.049	0.062	0.055	0.045	0.062	0.058	1	0

**Table 3:** The weight and the ranking of VGAS criteria for grades 5 and 6.

	Q1	Q2	Q3	Q4	Q5	Q6	Q7	Q8	Q9	Q10	Q11	Q12	Q13	Q14	Q15	Q16	Q17	Q18	Sum	CI
Grade 5																				
DM1	0.050	0.057	0.064	0.057	0.050	0.050	0.057	**0.089**	**0.068**	0.050	0.050	0.053	0.050	0.053	0.050	0.050	0.053	0.050	1	0
DM2	0.044	0.061	0.073	0.054	0.041	0.041	0.063	**0.096**	0.068	0.055	0.042	0.052	0.050	0.068	0.048	0.045	0.053	0.045	1	0
DM3	0.037	0.049	0.039	0.047	**0.066**	**0.070**	0.055	0.056	0.059	0.060	0.055	0.056	0.055	**0.070**	**0.066**	0.054	0.055	0.051	1	0
DM4	0.000	0.000	0.000	0.000	0.000	0.000	0.000	0.000	0.000	0.000	0.000	0.000	0.000	0.000	0.000	0.000	0.000	0.000	--	--
Weight	0.043	0.058	0.069	0.054	0.045	0.045	0.061	0.092	0.067	0.055	0.044	0.054	0.051	0.067	0.050	0.046	0.053	0.046	1	0
Grade 6																				
DM1	0.051	0.054	0.072	0.054	0.048	0.048	0.057	**0.102**	0.051	0.051	0.048	0.048	0.048	0.075	0.048	0.048	0.051	0.048	1	0
DM2	0.053	0.059	**0.085**	0.054	0.042	0.042	0.061	**0.084**	0.069	0.052	0.041	0.053	0.045	0.070	0.050	0.042	0.056	0.042	1	0
DM3	0.065	0.065	0.062	0.035	0.047	0.062	0.057	0.045	0.060	0.059	0.062	0.062	0.052	**0.082**	0.040	0.042	0.050	0.050	1	0
DM4	0.000	0.000	0.000	0.000	0.000	0.000	0.000	0.000	0.000	0.000	0.000	0.000	0.000	0.000	0.000	0.000	0.000	0.000	--	--
Weight	0.054	0.059	0.080	0.051	0.043	0.045	0.060	0.081	0.065	0.053	0.045	0.054	0.046	0.072	0.048	0.043	0.055	0.044	1	0

**Table 4:** The weight and ranking of VGAS criteria for middle school students (grades 7, 8, and 9).

	Q1	Q2	Q3	Q4	Q5	Q6	Q7	Q8	Q9	Q10	Q11	Q12	Q13	Q14	Q15	Q16	Q17	Q18	Sum	CI
Grade 7																				
DM1	0.000	0.000	0.000	0.000	0.000	0.000	0.000	0.000	0.000	0.000	0.000	0.000	0.000	0.000	0.000	0.000	0.000	0.000	--	--
DM2	0.055	0.075	0.081	0.049	0.038	0.045	0.064	**0.103**	0.062	0.049	0.042	0.054	0.042	0.081	0.039	0.040	0.044	0.036	1	0.001
DM3	0.063	**0.082**	0.072	0.061	0.022	0.048	0.069	0.067	0.067	0.052	0.052	0.069	0.046	0.067	0.037	0.039	0.041	0.045	1	0
DM4	0.060	**0.075**	**0.075**	0.030	0.015	0.030	**0.075**	0.060	0.060	0.060	0.060	0.060	0.060	0.060	0.030	0.060	**0.075**	0.060	1	0
Weight	0.056	0.076	0.079	0.050	0.035	0.045	0.064	0.098	0.063	0.051	0.044	0.056	0.043	0.079	0.039	0.040	0.044	0.037	1	0
Grade 8																				
DM1	0.052	0.052	0.061	0.056	0.047	0.047	0.066	**0.089**	0.066	0.047	0.047	0.047	0.047	**0.070**	0.047	0.047	0.061	0.054	1	0.001
DM2	0.052	0.066	**0.087**	0.050	0.044	0.040	0.057	**0.093**	0.069	0.054	0.043	0.047	0.049	0.071	0.045	0.040	0.052	0.041	1	0
DM3	**0.067**	**0.067**	**0.074**	0.060	0.043	0.051	0.054	0.047	0.062	0.054	0.047	0.061	0.054	0.058	0.057	0.041	0.051	0.051	1	0
DM4	0.052	**0.067**	**0.067**	0.037	0.045	0.052	0.052	**0.074**	0.052	0.052	0.037	0.052	0.052	**0.067**	0.060	0.060	0.060	0.062	1	0
Weight	0.055	0.066	0.083	0.052	0.044	0.043	0.057	0.084	0.068	0.054	0.043	0.050	0.050	0.068	0.048	0.041	0.052	0.042	1	0
Grade 9																				
DM1	0.047	**0.094**	0.062	0.062	0.047	0.047	0.047	0.062	0.062	0.048	0.047	0.062	0.047	**0.078**	0.047	0.047	0.047	0.047	1	0
DM2	0.051	**0.072**	**0.079**	0.053	0.043	0.045	0.059	**0.088**	0.065	0.053	0.040	0.056	0.054	**0.074**	0.041	0.046	0.046	0.036	1	0
DM3	0.057	**0.071**	0.068	0.051	0.048	0.057	0.060	0.063	0.062	0.049	0.041	0.056	0.056	**0.076**	0.045	0.050	0.049	0.041	1	0
DM4	0.059	**0.070**	0.059	0.059	0.056	0.056	0.059	0.063	**0.070**	0.059	0.059	**0.070**	0.059	0.059	0.038	0.031	0.028	0.045	1	0
Weight	0.054	0.072	0.074	0.054	0.046	0.050	0.059	0.077	0.064	0.052	0.042	0.057	0.055	0.073	0.042	0.046	0.045	0.038	1	0

**Table 5:** The weight and the ranking of VGAS criteria for high school students (grades 10, 11, and 12).

	Q1	Q2	Q3	Q4	Q5	Q6	Q7	Q8	Q9	Q10	Q11	Q12	Q13	Q14	Q15	Q16	Q17	Q18	Sum	CI
Grade 10																				
DM1	0.050	0.050	**0.100**	0.050	0.050	0.050	0.050	0.050	0.050	0.050	0.050	0.050	**0.100**	0.050	0.050	0.050	0.050	0.050	1	0
DM2	0.048	**0.077**	**0.086**	0.050	0.048	0.039	0.058	**0.085**	0.060	0.043	0.034	0.061	0.055	**0.076**	0.039	0.042	0.058	0.041	1	0
DM3	0.047	**0.069**	**0.075**	0.056	0.043	0.052	0.049	0.063	0.055	0.062	0.044	0.058	0.058	**0.076**	0.046	0.040	0.058	0.050	1	0.001
DM4	**0.070**	**0.070**	0.042	**0.070**	**0.070**	0.056	0.063	0.042	0.056	0.021	0.063	**0.070**	**0.070**	0.049	0.049	0.063	0.042	0.028	1	0
Weight	0.049	0.074	0.081	0.053	0.047	0.044	0.055	0.076	0.058	0.049	0.039	0.060	0.057	0.075	0.042	0.042	0.057	0.044	1	0
Grade 11																				
DM1	0.049	0.049	0.049	0.049	0.049	0.049	0.049	0.049	0.049	0.049	0.049	0.073	**0.098**	0.073	0.049	0.073	0.049	0.049	1	0
DM2	0.052	**0.072**	**0.079**	0.052	0.049	0.048	0.060	**0.086**	0.062	0.053	0.039	0.053	0.048	**0.077**	0.041	0.046	0.045	0.038	1	0
DM3	0.050	**0.068**	0.056	0.058	0.060	0.057	0.057	**0.068**	0.051	0.052	0.051	0.048	0.051	**0.065**	0.046	0.057	0.058	0.047	1	0
DM4	0.045	**0.065**	0.060	**0.065**	0.057	0.058	0.045	0.026	0.058	0.059	0.058	0.058	0.052	**0.065**	**0.065**	0.058	0.052	0.052	1	0
Weight	0.051	0.071	0.074	0.055	0.052	0.051	0.058	0.081	0.058	0.052	0.040	0.052	0.050	0.073	0.043	0.050	0.050	0.039	1	0
Grade 12																				
DM1	0.000	0.000	0.000	0.000	0.000	0.000	0.000	0.000	0.000	0.000	0.000	0.000	0.000	0.000	0.000	0.000	0.000	0.000	--	--
DM2	0.048	**0.079**	**0.076**	0.050	0.051	0.046	0.062	**0.089**	0.063	0.054	0.035	0.058	0.057	0.066	0.036	0.045	0.046	0.040	1	0
DM3	0.046	**0.077**	**0.075**	0.058	0.047	0.056	0.053	0.066	0.057	0.057	0.038	0.060	**0.069**	0.065	0.041	0.041	0.056	0.039	1	0
DM4	0.031	0.065	**0.069**	0.046	0.046	0.046	0.065	0.065	0.038	0.034	0.046	0.053	0.065	**0.069**	0.057	**0.069**	**0.069**	**0.069**	1	0
Weight	0.046	0.077	0.074	0.052	0.049	0.049	0.060	0.080	0.059	0.052	0.037	0.057	0.060	0.065	0.040	0.047	0.051	0.042	1	0

**Table 6:** Ranking the alternatives prone to gaming.

	*Db_i_*	*dw_i_*	*R_i_*	Rank		*db_i_*	*dw_i_*	*R_i_*	Rank		*db_i_*	*dw_i_*	*R_i_*	Rank
Grade 3					Grade 4					Grade 5				
DM1	0.206	0	0	3	DM1	0.188	0	0	3	DM1	0.202	0.007	0.037	2
**DM2**	0	0.206	1	**1**	**DM2**	0	0.188	1	**1**	**DM2**	0	0.208	1	**1**
DM3	0.174	0.034	0.165	2	DM3	0.108	0.085	0.441	2	DM3	0.205	0.003	0.018	3
Grade 6														
DM1	0.196	0.007	0.036	3										
**DM2**	0	0.199	1	**1**										
DM3	0.190	0.012	0.059	2										
Grade 7					Grade 8					Grade 9				
--					DM1	0.219	0.006	0.027	3	DM1	0.203	0	0	4
**DM2**	0	0.237	1	**1**	**DM2**	0	0.224	1	**1**	**DM2**	0	0.203	1	**1**
DM3	0.200	0.034	0.158	2	DM3	0.180	0.047	0.207	2	DM3	0.096	0.109	0.531	2
DM4	0.237	0	0	3	DM4	0.224	0	0	4	DM4	0.178	0.026	0.129	3
Grade 10					Grade 11					Grade 12				
DM1	0.210	0	0	4	DM1	0.212	0	0	4	--				
**DM2**	0	0.210	1	**1**	**DM2**	0	0.212	1	**1**	**DM2**	0	0.185	1	**1**
DM3	0.100	0.113	0.529	2	DM3	0.114	0.101	0.470	2	DM3	0.116	0.071	0.381	2
DM4	0.201	0.010	0.051	3	DM4	0.203	0.010	0.046	3	DM4	0.185	0	0	3

**Table 7:** Overall VGAS criteria ranking across the grades.

	Q1	Q2	Q3	Q4	Q5	Q6	Q7	Q8	Q9	Q10	Q11	Q12	Q13	Q14	Q15	Q16	Q17	Q18
Grade 3	0.390	0.395	0.392	0.414	0.411	0.421	0.406	0.390	0.400	0.402	0.409	0.400	0.404	0.396	0.410	0.407	0.395	0.418
Rank	**1**	4	**3**	16	15	18	11	**2**	8	9	13	7	10	6	14	12	5	17
Grade 4	0.398	0.388	0.384	0.398	0.388	0.395	0.389	0.376	0.387	0.388	0.393	0.397	0.392	0.384	0.395	0.387	0.388	0.395
Rank	18	7	**3**	17	9	15	10	**1**	5	6	12	16	11	**2**	13	4	8	14
Grade 5	0.389	0.386	0.384	0.391	0.406	0.407	0.389	0.387	0.390	0.393	0.400	0.397	0.393	0.389	0.399	0.396	0.393	0.396
Rank	5	**2**	**1**	8	17	18	4	**3**	7	10	16	14	11	6	15	13	9	12
Grade 6	0.397	0.394	0.387	0.384	0.405	0.405	0.390	0.394	0.386	0.395	0.406	0.394	0.396	0.400	0.386	0.402	0.388	0.397
Rank	12	7	4	**1**	17	16	6	8	**3**	10	18	9	11	14	**2**	15	5	13
Grade 7	0.388	0.387	0.382	0.389	0.375	0.386	0.387	0.377	0.386	0.386	0.390	0.391	0.387	0.381	0.383	0.385	0.385	0.392
Rank	14	11	4	15	**1**	8	12	**2**	9	10	16	17	13	**3**	5	7	6	18
Grade 8	0.435	0.440	0.420	0.469	0.475	0.481	0.462	0.419	0.417	0.467	0.459	0.421	0.454	0.424	0.442	0.461	0.434	0.436
Rank	7	9	**3**	16	17	18	14	**2**	**1**	15	12	4	11	5	10	13	6	8
Grade 9	0.463	0.446	0.433	0.448	0.469	0.480	0.452	0.419	0.446	0.443	0.462	0.454	0.456	0.446	0.454	0.450	0.450	0.463
Rank	16	6	**2**	7	17	18	10	**1**	4	**3**	14	12	13	5	11	9	8	15
Grade 10	0.441	0.430	0.425	0.453	0.433	0.472	0.426	0.415	0.433	0.470	0.470	0.435	0.445	0.437	0.456	0.439	0.437	0.456
Rank	11	4	**2**	13	6	18	**3**	**1**	5	17	16	7	12	9	14	10	8	15
Grade 11	0.426	0.425	0.416	0.441	0.447	0.445	0.424	0.410	0.416	0.430	0.437	0.422	0.430	0.417	0.443	0.449	0.453	0.449
Rank	8	7	**2**	12	15	14	6	**1**	**3**	9	11	5	10	4	13	16	18	17
Grade 12	0.435	0.436	0.436	0.450	0.430	0.460	0.433	0.417	0.427	0.432	0.449	0.438	0.458	0.437	0.470	0.449	0.467	0.456
Rank	6	7	8	13	**3**	16	5	**1**	**2**	4	11	10	15	9	18	12	17	14

**Table 8:** The level of gaming among the students of grade 3 to grade 12.

Grades	DM1 No Symptom	DM2 At Risk	DM3 Have Some Levels of Addiction	DM4 Already Addicted
Grade 12	--	71.83%	22.53%	5.63%
Grade 11	2%	72%	24%	2%
Grade 10	1%	71%	26%	2%
Grade 9	3%	67%	25%	5%
Grade 8	7.93%	79.36%	11.11%	1.58%
Grade 7	--	89%	10%	1%
Grade 6	18%	73%	9%	--
Grade 5	17.07%	76.83%	6.1%	--
Grade 4	11.32%	67.92%	20.75%	--
Grade 3	12.12%	75.75%	12.12%	--

## Data Availability

The datasets that were used and analyzed in the study are available from the corresponding author on reasonable request.

## Appendix A

1. Do you find it difficult to stop playing video games?	Never	Rarely	Sometimes	Often	Always
2. Have you played extra video games longer than intended?					
3. Have you had arguments with others (family and friends) over your time spent on video games?					
4. How often do you prefer to play video games instead of spending time with others?					
5. How often do you not get enough sleep because of playing video games?					
6. Have you thought all day long or when you wake up in the morning, about playing video game?					
7. How often do you look forward to the next time you can play video games?					
8. How often do you think you should play less video game?					
9. How often have you unsuccessfully tried to spend less time on playing video games?					
10. Have others unsuccessfully tried to reduce your time spent on video games?					
11. How often do you feel upset, restless, frustrated, or irritated when you were UNABLE to spend time for playing video games?					
12. How often do you rush through your daily responsibilities to play video games?					
13. Have you neglected important activities (school, work, and sports) to play video games?					
14. How often do you play video games because you are feeling down or to forget about problems?					
15. Since last semester, I have spent more time the playing video game every week than before.					
16. I used to have backache or other physical discomforts because of playing video games.					
17. Playing video games has caused some negative effects on my study.					
18. Without playing video games, my life would be no fun.					
